# Ultra-high field neuroimaging in psychosis: A narrative review

**DOI:** 10.3389/fpsyt.2022.994372

**Published:** 2022-11-24

**Authors:** Katie M. Lavigne, Kesavi Kanagasabai, Lena Palaniyappan

**Affiliations:** ^1^Douglas Mental Health University Institute, McGill University, Montreal, QC, Canada; ^2^Montreal Neurological Institute-Hospital, McGill University, Montreal, QC, Canada; ^3^Department of Psychiatry, McGill University, Montreal, QC, Canada; ^4^Robarts Research Institute, Western University, London, ON, Canada; ^5^Department of Medical Biophysics, Western University, London, ON, Canada

**Keywords:** schizophrenia, psychosis, ultra-high field neuroimaging, 7 tesla, magnetic resonance spectroscopy, magnetic resonance imaging, multiscale

## Abstract

Schizophrenia and related psychoses are complex neuropsychiatric diseases representing dysconnectivity across multiple scales, through the micro (cellular), meso (brain network), manifest (behavioral), and social (interpersonal) levels. *In vivo* human neuroimaging, particularly at ultra-high field (UHF), offers unprecedented opportunity to examine multiscale dysconnectivity in psychosis. In this review, we provide an overview of the literature to date on UHF in psychosis, focusing on microscale findings from magnetic resonance spectroscopy (MRS), mesoscale studies on structural and functional magnetic resonance imaging (fMRI), and multiscale studies assessing multiple neuroimaging modalities and relating UHF findings to behavior. We highlight key insights and considerations from multiscale and longitudinal studies and provide recommendations for future research on UHF neuroimaging in psychosis.

## Multiscale dysconnectivity in psychosis

Schizophrenia, coined term for “split mind,” is a complex neuropsychiatric illness characterized by a range of positive, negative, and cognitive symptoms (1). Positive symptoms include hallucinations (false perceptions) and delusions (false beliefs) that are often characteristic of psychotic disorders. Negative symptoms refer to a decrease in typical behaviors, such as reduced motivation, pleasure, speech and expressed affect. Finally, cognitive symptoms manifest as impaired performance across a range of neuropsychological domains, including language, memory, and attention. These symptoms are evident in psychosis, an umbrella term that groups individuals diagnosed with schizophrenia and related psychotic disorders (e.g., schizoaffective disorder, schizophreniform disorder, bipolar disorder with psychotic features, etc.).

**FIGURE 1:**
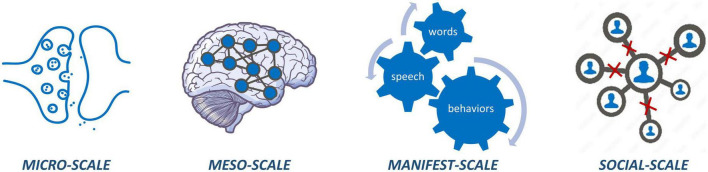
Micro-scale dysconnectivity observed at synapses and circuits; meso-scale dysconnectivity across brain networks; manifest-scale dysconnectivity between how words are put together, actions are put together, and memories are put together; social-scale carries dysconnectivity at various interpersonal relations hips.

Psychotic disorders can be understood as disorders of connectivity across multiple scales, ranging from micro (cellular level) to meso (brain network level) to manifest (behavioral level) to social (interpersonal level) scales (Figure 1). At the microscale, psychosis is associated with dysfunctional glutamatergic and dopaminergic transmission across synapses as well as altered astrocyte signaling (2, 3). At the meso-scale, psychosis is characterized by both structural and functional dysconnectivity of brain networks (4–6). Behaviorally, individuals with psychosis manifest a wide range of symptoms that point toward dysfunction in relational processing (e.g., relational memory, social cognition, linguistic processing), affecting interpersonal relationships at the social scale (7–9). Thus, psychosis does not arise from abnormality in any single point source but likely results from a sum of aberrant interactions between networks of features within and across scales.

Beyond identifying dysconnectivity at multiple scales, a complete picture of psychosis is only possible through understanding the way in which these scales interact and influence one another. For example, micro-level dysfunction (e.g., aberrant concentrations of neural metabolites and neurotransmission), may present itself in meso-level dysfunction (e.g., brain dysconnectivity), which manifests as abnormal speech and behavioral patterns that affect the interpersonal relationships of individuals living with schizophrenia (10). Multiscale studies incorporating measures from the micro to social scale are, therefore, of critical importance to connect social and behavioral findings with cellular and network level disruptions in psychosis. *In vivo* human neuroimaging can provide insight into multiscale dysconnectivity at the microscale (e.g., neurotransmitter dysfunction through magnetic resonance spectroscopy; MRS; and positron emission tomography; PET) and mesoscale (e.g., structural and functional dysconnectivity through magnetic resonance imaging; MRI), while simultaneously allowing for acquisition of data at the manifest (e.g., behavioral, clinical, cognitive) and social (e.g., interpersonal) scales.

Ultra-high field (UHF) neuroimaging (e.g., 7T MRI/MRS) refers to magnetic resonance imaging using a magnetic field strength (b0) of greater than or equal to 7.0 Tesla. UHF offers many benefits relative to lower fields strengths (e.g., 3T) that can facilitate multiscale dysconnectivity research in psychiatric populations (11, 12). Quantitative improvements of UHF include greater signal-to-noise (SNR) and contrast-to-noise ratios and higher spatial resolution. This leads to qualitative advantages over lower field strengths, such as the ability to visualize smaller structures and layer-level structural and functional dysconnectivity. UHF-MRS also presents qualitative and quantitative improvements over standard MRS, as we discuss below, such as better differentiation and identification of previously undetectable metabolites at the microscale. Increased sensitivity and resolution translate to greater power to detect effects in smaller samples, as well as at the individual level, giving impetus to precision psychiatry (13, 14). In this review, we provide an overview of the literature to date on UHF in psychosis, highlighting key insights and challenges of this work, and provide recommendations for future research. We emphasize multiscale UHF studies in psychosis throughout, particularly those studies incorporating measures across the micro, meso, manifest, and social scales.

## Magnetic resonance spectroscopy

### Advantages of ultra-high field-magnetic resonance spectroscopy

A specific advantage offered by UHF-MRS systems is the ability to resolve the neurometabolic spectrum along with high SNR. The SNR gain from 3T to 7T is especially pronounced for glutamate, glutamine, and gamma aminobutyric acid (GABA). Glutamine and glutamate are molecules in a dynamic equilibrium, reflecting the integrity of distinct cellular compartments (neurons and astrocytes) in the brain, but separating their spectra is challenging at lower field strengths. The reduced error rates in quantifying these molecules at UHF translates to a reduced need for large sample sizes to observe a given effect. This is especially helpful in the study of schizophrenia where neurobiological heterogeneity is a prime suspect for poor therapeutic progress.

UHF proton MRS (^1^HMRS) has overcome several methodological limitations evident at lower field strengths, such as separating highly coupled spins, improving SNR of metabolites with low *in vivo* concentrations, increased spectral dispersion, lower uncertainty in spectral quantification such as smaller Cramer lower bounds (CRLB) and coefficients of variance (CV). For example, glutathione is typically isolated as a secondary metabolite to GABA at lower field strengths using a single shot with spectral editing which can lead to inconsistent results (15), whereas at 7T, glutathione can be more reliably detected and separated from other metabolites. Specialized pulse sequences, a series of gradients and radiofrequency (RF) pulses, required to isolate highly coupled metabolites at lower field strengths are not necessary with UHF-^1^HMRS as it is better able to resolve structurally similar metabolites such as glutamate and glutamine. Furthermore, UHF-^1^HMRS resolves the glutamate signals that contaminate GABA and glutathione peaks at clinically feasible scan times for many brain regions, producing improved spectral outputs for GABA and glutathione when assessing the pathology of psychiatric illnesses (16).

### Key findings

A major working hypothesis regarding synapse-level dysconnectivity in psychosis is centered around three neurotransmitters: dopamine, glutamate and GABA. Of these, the relatively higher concentration and wider distribution of glutamate and GABA compared to dopamine (17) makes them suitable candidates for quantification using ^1^HMRS. Currently there are 17 published articles reporting metabolites in patients who are at a pre-psychotic (clinical high risk) stage, experiencing first episode psychosis (FEP), or with established schizophrenia using UHF-^1^HMRS (Table 1).

**TABLE 1:** Ultra-high field magnetic resonance spectroscopy/magnetic resonance spectroscopic imaging findings.

Study	Dx	N patients	Region(s) and voxel size (mm)	Pulse sequence	Parameters (ms)	Main findings
Brandt et al. (65)	SZ	24	ACC: 30 × 20 × 12	STEAM	TE_1/2_: 14/28 TR: 3,000	– ACC GLU SZ < HC – ACC GLU decreased with age in SZ
Broeders et al. (25)	SZ	16	DGM: 5 × 5 × 10	FID	TE: 2.5 TR: 300	– DGM GLU SZ < HC – Low DGM GLU lower psychomotor speed
Dempster et al. (28)*	SZ	26	dACC: 20 mm iso	Semi–LASER	TE: 100 TR: 7,500	– High dACC GSH shorter treatment response – High dACC GLU lower functioning
Godlewska et al. (29)	FEP	17	ACC: 20 mm iso (DLPFC and PUT)	STEAM	TE: 11 TR: 5,000	– ACC GLU and Gln FEP < HC – High ACC GLU higher cognition in FEP
Jeon et al. (37)	CHR	13	dACC: 20 mm iso	Semi–LASER	TE: 100 TR: 7,500	– dACC GSH CHR > HC – High ACC GSH higher functioning in CHR
Kumar et al. (35)	SZ	28	ACC: 20 × 18 × 25 (INS and OCC)	Short TE STEAM	TE: 17 TM: 17 TR: 2,000	– ACC, INS and OCC GLU andGSH SZ < HC
Mackinley et al. (34)^#^	FEP	57	dACC: 20 mm iso	Semi–LASER	TE: 100 TR: 7,500	– Baseline dACC GSH FEP in employment, education by 1–year > HC = FEP not in employment/education
Pan et al. (33)^#^	FEP	40	dACC: 20 mm iso	Semi–LASER	TE: 100 TR: 7,500	– dACC GSH SZ high CD > SZ low CD – High GSH lower cingulum FA in SZ high CD
Posporelis et al. (66)	FEP	20	ACC: 30 mm iso	STEAM	TE: 15 TM: 25 TR: 3,000	– Normative association b/w brain temperature and ACC GLU not present in FEP
Quiñones et al. (23)	CHR	15	(Whole brain) Thalamus: 9 × 9 × 10	J–RCT	TE: 34 TR: 1,500	– Thalamus GABA/GLU CHR < HC – Low GABA/GLU higher Sx in CHR
Reid et al. (22)	SZ	23	ACC: 27 × 20 × 10	Ultra– short TE STEAM	TE: 5 TM: 45 TR: 10,000	– ACC GLU/NAA SZ > HC – High GABA lower cognition in SZ
Roalf et al. (26)	CHR SZ	19	Subcortex/lobular FOV: 220 × 200 Matrix: 192 × 192	GLU–CEST	TE: 3 TR_GRE_: 6.2 TR: 10,500	– Subcortical/lobular GLU SZ/CHR < HC – Low frontal GLU higher negative Sx – Low parietal GLU higher positive Sx
Rowland et al., (38)	SZ	27	ACC: 30 × 20 × 20	STEAM	TE: 14 TM: 33 TR: 3,000	– Cerebral lactate SZ > HC – High lactate lower cognition and functioning
Thakkar et al. (19)	SZ	21	BG, OCC: 40 × 24 × 25	MEGA–/semi–LASER	TE: 36/74 TR: 5,000	– OCC GABA SZ < HC/REL – OCC GLN SZ/REL < HC
Wang et al. (20)	FEP	81	dACC: 20 × 30 × 20 CSO: 20 × 40 × 15 DLPFC: 20 × 25 × 20 OFR: 20 mm iso Thalamus: 30 × 20 × 15	STEAM	TE: 14 TM: 33 TR: 3,000	– dACC GLU/GABA/GSH, OFR NAA, thalamus NAA/GSH FEP < HC – High NAA CSO white matter higher cognitive performance
Wijtenburg et al. (18)	SZ	40	ACC: 30 × 20 × 20 CSO: 40 × 20 × 15 DLPFC: 25 × 20 × 20 Hipp: 35 × 15 × 15 Thalamus: 20 × 30 × 15	STEAM	TE: 14 TM: 33 TR: 3,000	– ACC GLU SZ < HC/REL – High ACC GLU higher functioning and cognition – CSO Gln/GLU SZ ≤ REL ≤ HC – High CSO Gln/GLU lower functioning – Sx associations differed by illness duration
Yang et al. (30)	FEP	136	ACC: 20 × 30 × 20 (CSO, DLPFC, OFR, Thalamus)	STEAM	TE: 14 TM: 33 TR: 3,000	–ACC GSH FEP treatment resistant < non–treatment resistant

AI, anterior insula; BG, basal ganglia; C, cross-sectional; CD, conceptual disorganization; CHR, clinical high risk; CSO, centrum semiovale; d/ACC, (dorsal) anterior cingulate cortex; DGM, deep gray matter; DLPFC, dorsolateral prefrontal cortex, Dx, diagnosis; FEP, first-episode psychosis; FES, first-episode schizophrenia; FID, free induction decay; FOV, field of view; GABA, gamma amino butyric acid; Gln, glutamine; GLU, glutamate; GSH, glutathione; HC, healthy control; INS, insula; J-RCT, J-refocused coherence transfer; L, longitudinal; NAA, N-acetylaspartate; OCC, occipital cortex; OFR, orbitofrontal region; PUT, putamen; REL, relatives; semi-LASER, semi-localized by adiabatic selective refocusing; SFG, superior frontal gyrus; STEAM, stimulated echo acquisition mode; Sx, symptoms; SZ, schizophrenia; TE, echo time; TM, mixing time; TR, repetition time; *, longitudinal; ^#^, overlapping samples.

Most of these studies report on GABA and glutamate. Wijtenburg et al. (18) assessed glutamine, glutamate, GABA, and lactate differences among patients with schizophrenia, first-degree relatives, and healthy controls in five brain regions and reported glutamate differences in the anterior cingulate cortex (ACC); GABA, glutamine, and glutamate differences in the centrum semiovale; and no metabolite differences in the dorsolateral prefrontal cortex, hippocampus, or thalamus. Thakkar et al. (19) similarly studied patients, relatives and unrelated healthy controls, reporting a reduction in GABA and glutamate among patients. A reduction in GABA was also reported by Wang et al. (20) across several brain regions (ACC, orbitofrontal cortex, and thalamus) and by Marsman et al. (21) in the prefrontal cortex. This reduction in a network of metabolites is significantly correlated with severity of cognitive deficits among patients living with first episode schizophrenia. Reid et al. (22) found a correlation between GABA levels and cognitive function among patients with schizophrenia. GABA reduction may precede symptom onset, becoming apparent in the thalamus even at the clinical high-risk state (23).

While the ACC has been the most preferred site of voxel placement for 7T MRS studies in psychosis to date (24), a more recent study using a lipid suppression coil focused on deeper gray matter structures (2D magnetic resonance spectroscopic imaging, MRSI), reporting a generalized glutamate reduction in medicated patients that related to their psychomotor speed (25). Using a more spatially resolved 2D approach for glutamate quantification based on chemical exchange saturation (GluCEST), Roalf et al. (26) noted an overall decline in glutamate levels in patients with early stage psychosis. The advantage of GluCEST is the ability to quantify regional brain glutamate distribution across multiple cross-sectional whole brain slices in a reasonably short time period (27), thus promising more extensive studies of glutamate physiology in the near future.

Besides glutamate, glutamine and GABA, several studies have investigated glutathione—a critical marker of healthy neuro-glial interaction (20, 28–31) (see Table 1). In general, there is a higher degree of variability in cortical glutathione concentration among patients (32), with a subset showing higher glutathione levels and distinct patterns of clinical symptoms during first episode of psychosis (33), with better outcomes over time (28), especially in relation to their functional trajectory (34). In contrast, those with pronounced reduction in glutathione may have higher residual symptom burden (35). Limongi et al. (36) proposed that higher glutathione levels could reverse the glutamate-mediated dysconnectivity based on resting-state functional MRI-based causal modeling in combination with 7T MRS. In line with this, youth who are at a high risk for psychosis have higher glutathione in association with superior social functioning (37, 38).

### Key insights and future directions in ultra-high field magnetic resonance spectroscopy

UHF-^1^HMRS has greatly facilitated the detection of multiple metabolites in a single spectrum and the study of multiscale associations between metabolites and clinical measures. While many studies agree with prior findings at 3T; making direct comparisons across different studies is challenging due to differences in pulse sequences used to isolate and suppress signal, different quantification, fitting, tissue correction, water suppression techniques, and different preference of reference metabolites. Although these differences are small within a single study, there is a growing area of ^1^HMRS development that accounts for these differences (39). This could aid in the process to develop a uniform protocol to assess the quality of reported metrics for future studies to enable targeted treatment based on potential biomarkers.

Although macromolecule contamination does not appear to vary among patients and healthy controls, it may still affect the estimations of glutamate, GABA, and glutamine which could affect the interpretation of the pathophysiology of metabolites. The MRS pulse sequence and field strength used to detect and isolate metabolite(s) of interest could help eliminate and/or better estimate the effects of macromolecule contaminations. At lower field strengths, especially with the use of spectral editing, studies report GABA as GABA+ to indicate the macromolecule contamination. Using higher field strengths, high order shimming and local power optimization is crucial to minimize field-dependent inhomogeneities and to ensure accurate RF pulse frequency profiles. Inaccurate RF pulse profiles are common among pulses with larger flip angles such as 180° and to achieve the exact 180° flip can be difficult resulting in inaccurate isolation of metabolites. Furthermore, with increasing field strengths, transverse (T2) relaxation times and J-modulation effects decrease at a faster rate relative to lower field strengths. Hence, the quality of results is dependent on MRS pulse sequence use and effective echo times to ensure better detection. In the case of using a long echo time to ensure detection of GABA, 7T poses higher specific absorption rate, also known as tissue heating, when using a continuous pulse along with signal decay with shorter T2 relaxations. This poses a problem when detecting internal references such as creatine and water. Sequences have become robust to target signal loss via shorter echo times, however, the quality of data using CRLB or CV have reported higher than the accepted 20% threshold.

To date, MRS has been the most studied modality with UHF neuroimaging in psychosis, and many of these studies have investigated multiscale associations at two or more levels, from the microscale to the social scale. Though important differences are detectable at smaller sample sizes, future studies with larger samples can ensure representativeness and allow for investigation of potential mediating/moderating effects of sociodemographic (e.g., age, sex, gender) and clinical (e.g., psychosis stage, comorbidities) variables. Conducting longitudinal research across multiple scales will be essential to understand the therapeutic utility of MRS markers in the long term.

## Structural magnetic resonance imaging

### Advantages of ultra-high field-structural magnetic resonance imaging

UHF also confers both quantitative and qualitative advantages to lower field strengths in terms of brain structure. One of the most promising applications of ultra-high field structural magnetic resonance imaging (UHF-sMRI) is the potential to non-invasively image the laminar structure of the brain *in vivo*, which is not possible at lower field strengths. In addition, UHF-sMRI can also more clearly visualize smaller structures and subregions. A prime example relevant to psychiatric applications is the hippocampus, its subfields, and adjacent white matter output regions, which are strongly implicated in schizophrenia (40, 41). Quantitatively, UHF-sMRI can also provide a better delineation of the gray-white matter boundary, which improves tissue segmentation and accuracy of cortical metrics, such as cortical thickness and surface area. Finally, higher image resolution can mitigate partial volume effects due to smaller voxel sizes. Together, these advantages improve precision and sensitivity of structural MRI at UHF, allowing for more detailed mesoscale and multiscale investigations.

### Key findings

To date, 11 studies have assessed brain structure using UHF-sMRI in schizophrenia-spectrum disorders, including schizophrenia, schizoaffective disorder, first-episode psychosis, and childhood-onset schizophrenia (Table 2). Iwabuchi et al. (42) compared machine learning classification of gray matter and white matter intensity images acquired at 3T and 7T, showing improved accuracy, sensitivity, and specificity of patient classification at 7T. Reduced hippocampus and hippocampal subfield volumes are a robust finding at lower field strengths in schizophrenia-spectrum disorders (43, 44). These findings have been replicated at 7T in first-episode psychosis (41) and childhood-onset schizophrenia (45), with the latter reporting multiscale associations with cognitive impairment. In an innovative study highlighting the qualitative improvements of UHF-sMRI, Kirov et al. (46) visualized the granule cell layer of the hippocampal dentate gyrus at 232 × 232 × 1,500 μm in 16 schizophrenia patients and 15 healthy controls. They observed that lower neuroradiologist-rated intensity was related to diagnosis, but not to symptom severity or disease duration.

**TABLE 2:** Ultra-high field structural magnetic resonance imaging findings.

Study	Dx	N	Structural measure	Pulse sequence	Voxel size (mm)	Coverage	Main findings
Hua et al. (67)	SSD	12	CBV	iVASO TFE	3.5 × 3.5 × 5	Whole brain	–Distributed decreases and increases in SZ vs. HC
Iwabuchi et al. (42)	SZ	19	GM and WM intensity	3T: MPRAGE 7T: IR-TFE	1 mm iso	Whole brain	Patient classification –7T > 3T –GM > WM
Kim et al. (50)	SZ	19	Serotonin transporter availability	7T: MPRAGE PET: [^11^C]DASB	0.8 mm iso	Whole brain (prefrontal ROIs)	–High prefrontal serotonin transporter availability lower insight
Kirov et al. (46)	SZ	16	Radiologist-rated intensity	MPRAGE-guided 2D GRE	232 × 232 × 1,500μm	Hippocampus DG cell layer	–DG intensity SZ < HC
Liang et al. (49)	FEP	25	Cortical thickness	MP2RAGE	0.8 mm iso	Whole brain	–Impoverishment subtype consistent with 3T samples –Impoverishment subtype higher dACC glutamate levels
Lottman et al. (68)	FES	19	GM, WM, and CSF volume	MPRAGE	0.7 mm iso	Whole brain	–Joint (GM + CSF, WM + ALFF) and unique modality-specific (WM, GM) components in FES
Palaniyappan et al. (48)^#^	SZ	17	MTR	MT-TFE	1 mm iso (3 mm iso resliced)	Whole brain (cingulum ROI)	–Cingulum MTR SZ < HC –High MTR higher delusions
Palaniyappan et al. (69)^#^	SZ	17	MTR	MT-TFE (7T)	1 mm iso (3 mm iso resliced)	Whole brain	–Occipitotemporal-adjacent MTR SZ < HC
Palaniyappan et al. (47)^#^	SZ	19	GM intensity	IR-TFE	0.6 mm iso (1 mm iso resliced)	Whole brain	–Impoverished thinking associated with multivariate morphometric components in SZ
Palaniyappan et al. (70)^#^	SZ	19	Cortical thickness	IR-TFE	0.6 mm iso (1 mm iso resliced)	Whole brain	–FPN and DMN thickness SZ < HC –Salience and FPN spatial incoherence SZ > HC –FPN thinning and salience incoherence associated with positive FTD –Language network thinning in positive FTD
Pan et al. (33)	FEP	31	FA and fiber tract volume	2D spin-echo EPI	2 mm iso	Whole brain	–Cingulum FA SZ low CD < SZ high CD –High GSH lower cingulum FA in SZ high CD
Park et al. (41)	FEP	41	Volume and shape	MP2RAGE	0.8 mm iso	Volume: whole-brain Shape: hippocampus	–Hippocampal volume (especially CA4) FEP < HC
Zhou et al. (45)	Child onset SZ	14	Volume and tensor-based morphometry	MPRAGE and T2*-weighted	0.7 mm iso and 0.5 mm iso	Hippocampal subfields	–Hippocampal subfield volumes and deformation SZ < HC –Associated with cognition

ALFF, amplitude of low frequency fluctuations; C, cross-sectional; CBV, cerebral blood volume; CD, conceptual disorganization; CSF, cerebrospinal fluid; DG, dentate gyrus; DMN, default-mode network; FA, fractional anisotropy; FEP, first-episode psychosis; FES, first-episode schizophrenia; FTD, formal thought disorder; FPN, frontoparietal network; GM, gray matter; GSH, glutathione; HC, healthy control; L, longitudinal; MTR, magnetization transfer ratio; ROI, region of interest SSD, schizophrenia-spectrum disorder; SZ, schizophrenia; WM, white matter; ^#^, overlapping samples.

Other UHF-sMRI findings highlight multiscale associations between psychotic symptoms and structural alterations in schizophrenia, particularly within the cingulum bundle and cingulate cortex. Palaniyappan et al. (47) reported associations between negative formal thought disorder (i.e., impoverished thought) and morphometric alterations in gray matter intensity in cingulate cortex as well as insula, striatum, precuneus, and frontotemporal regions. These authors later observed that decreased magnetization transfer ratio, a proxy for reduced myelin content, in the left cingulum bundle was associated with Schneiderian delusion severity (48). UHF diffusion-weighted MRI has also implicated the cingulum bundle in psychotic symptoms. Pan et al. (33) found associations between high conceptual disorganization and decreased fractional anisotropy in the cingulum as well as decreased fiber volumes extending from this seed region. More recently, Liang et al. (49) recovered a consistent subtype of patients with schizophrenia with marked and generalized cortical thinning; this subtype was noticeable even when a smaller sample was studied using 7T compared to 3T MRI, and was associated with higher 7T-MRS derived glutamate levels. In another novel application, Kim et al. (50) used a hybrid 7T MRI-PET to report prefrontal serotonin transporter distribution in relation to insight in schizophrenia.

### Key insights and future directions in ultra-high field-structural magnetic resonance imaging

UHF-sMRI studies in psychosis have transcended simple replication of findings observed at lower field strengths. Researchers now have the opportunity to visualize small brain structures at unprecedented resolutions *in vivo*, as exemplified by imaging of the hippocampus dentate granule cell layer (46). UHF-sMRI also yields better classification of patients and controls relative to lower field strengths, which will bolster efforts toward translational neuroscience and precision psychiatry.

Alongside these advantages, there are several considerations in structural UHF neuroimaging. Of particular relevance to psychiatry, UHF-sMRI is highly sensitive to motion artifacts, which are often more pronounced in psychiatric populations (51). Moreover, high-field strengths lead to increased susceptibility artifacts, especially within deep brain regions commonly the focus of psychiatric studies (e.g., hippocampus). Potential artifacts can counteract the benefits of UHF neuroimaging and should be minimized during both data acquisition (e.g., prospective motion correction) and processing (e.g., retrospective motion correction, gradient correction). Though UHF improvements in SNR allow for smaller samples to detect effects, most UHF-sMRI studies to date include less than 30 patients, which brings into question the representativeness of these findings. Future work in larger and more diverse samples (e.g., age, sex, gender, language, ethnicity, psychosis stage) are warranted. Larger samples would also allow for further investigation into potential confounding or moderating variables, including sociodemographics and clinical comorbidities. Incorporating these considerations into future work examining dysconnectivity and multiscale associations at the micro and social scales will build toward a broader picture of psychosis.

## Functional magnetic resonance imaging

### Advantages of ultra-high field-functional magnetic resonance imaging

UHF functional MRI (UHF-fMRI) enjoys the same advantages as UHF-sMRI in terms of increased spatial resolution, leading to smaller voxel sizes and, thus, reduced partial volume errors. This improves blood-oxygen-level-dependent signal estimates from smaller brain regions, particularly in subcortical structures, and allows for more precise estimates of brain connectivity. Moreover, UHF confers increased temporal resolution as well as shortened acquisition times in some cases, which allows for greater sensitivity and benefits participants with less time in the scanner, which is particularly relevant for those with psychiatric disorders, such as schizophrenia. Novel techniques, including direct imaging of neuronal activity (DIANA-fMRI) recently demonstrated in rodents (52), offer unprecedented potential to combine spatial and temporal resolution to make precise neurophysiological inferences using UHF-fMRI.

### Key findings

We identified 11 UHF-fMRI studies, including 8 resting-state and 3 task-based studies using either a Stroop or passive listening task (Table 3). The majority of these (*n* = 9) employed first-episode psychosis or first-episode schizophrenia samples. Resting-state findings included both increased and decreased connectivity patterns in psychosis relative to healthy controls (53, 54), consistent with findings at lower field strengths (5). During a passive listening task, Doucet et al. (55) reported auditory cortex hyperactivity and altered tonotopic organization in schizophrenia patients with a history of hallucinations. Multiscale studies have linked functional dysconnectivity to disease duration (54), positive symptom severity (56, 57), the Stroop effect (10, 58, 59), and glutamate/glutathione dysfunction through MRS (10, 36, 59). For example, Limongi et al. (10) identified associations between altered glutamatergic neurotransmission (microscale) and functional dysconnectivity (mesoscale) in FEP, which was associated with Stroop performance (manifest-scale) and social withdrawal (social-scale). More recently, Alonso-Sanchez et al. (60) demonstrated that a computational linguistic measure of similarity among adjacent words (word-to-word semantic vector relationship in spoken text generated outside the scanner) is increased in first-episode schizophrenia, and this abnormality is related to higher intrinsic inhibitory tone within the Broca’s area and semantic hub, parameterized using a spectral dynamic causal model of resting-state 7T fMRI acquisition.

**TABLE 3:** Ultra-high field functional magnetic resonance imaging findings.

Study	Dx	N patient	EPI Acquisition	Voxel size	Smoothing kernel	Task/Rest	Main findings
Alonso-Sánchez et al. (60)	FES	30	TR = 1,000 ms TE = 20 ms Flip angle = 30° Multi-band factor = 3 iPat = 3	2 mm iso	–	Rest	–Higher temporal-frontal intrinsic inhibitory connectivity relates to higher semantic similarity
Dey et al. (53)	FEP	25	TR = 1,000 ms TE = 20 ms Flip angle = 30° FOV (read, phase) = 208 mm, 100%	3 mm iso	4 mm	Rest (open)	–Right STG, insula, Heschl’s gyrus centrality FEP < HC –Medial superior parietal centrality highly disorganized FEP > less disorganized FEP
Doucet et al. (55)	SZ	16	TR = 2,000 ms TE = 25 ms Flip angle = 47° FOV = 222 × 222 mm	1.5 mm iso	4.5 mm	Passive listening	–Auditory cortex activity SZ > HC –Altered tonotopic organization in SZ
Gawne et al. (58)	FEP	17	TR = 3,000 TE = 28 Flip angle = 70° FOV = 200 × 200 mm	0.85 × 0.85 × 1.8 mm	5 mm	Stroop color-naming	–Distributed activity incongruent > congruent and FEP > HC –Stroop effect correlated with MEG alpha/theta ratio
Hua et al. (54)	SSD	14	TR = 2,000 ms TE = 22 ms Flip angle = 60°	2.5 mm iso	None	Rest	–Thalamic connectivity w/sensorimotor, parietal, and temporal SZ > HC –Thalamic connectivity w/frontal, cingulate, striatum, cerebellum SZ < HC –Associated w/disease duration –Comparable effect sizes to 3T
Limongi et al. (10)^#^	FEP	20	TR = 1,000 ms TE = 20 ms Flip angle = 30° FOV (read, phase) = 208 mm/100%	2 mm iso	4 mm	Rest	–Salience network dysconnectivity associated w/Stroop, dACC glutamate hypofunction, and social withdrawal
Limongi et al. (36)^#^	FES	19	TR = 1,000 ms TE = 20 ms Flip angle = 30° FOV = 208 mm	2 mm iso	4 mm	Rest	–High GSH higher dACC/AI inhibitory activity –High GLU lower dACC inhibitory activity
Limongi et al. (56)^#^	FEP	19	TR = 1,000 ms TE = 20 ms Flip angle = 30° FOV = 208 mm	2 mm iso	4 mm	Rest	–Delusional severity associated with decreased frontostriatal effective connectivity
Lottman et al. (68)	FES	19	TR = 3,000 ms TE = 28 ms Flip angle = 70° FOV = 200 mm	0.85 × 0.85 × 1.8 mm	5 mm	Rest	–Identified joint (GM + CSF, WM + ALFF) and unique modality-specific (WM, GM) components in early psychosis
Lottman et al. (57)	FES	19	TR = 3,000 ms TE = 28 ms Flip angle = 70° FOV = 200 mm	0.85 × 0.85 × 1.8 mm	5 mm	Rest	–Hyperconnectivity b/w subcortical and auditory networks, associated with symptoms (uncorrected) –Hypoconnectivity b/w interhemispheric sensorimotor sub-networks
Overbeek et al. (59)	FEP	17	TR = 3,000 ms TE = 28 ms Flip angle = 70° FOV = 200 mm	0.85 × 0.85 × 1.8 mm	5 mm	Stroop color naming	–Stroop effect widespread activation FEP > HC –Altered associations between GLU/GABA and BOLD signal in FEP

AI, anterior insula; BOLD, blood-oxygen-level-dependent; C, cross-sectional; Dacc, dorsal anterior cingulate cortex; FEP, first-episode psychosis; FES, first-episode schizophrenia; FOV, field of view; GABA, gamma amino butyric acid; GLU, glutamate; GSH, glutathione; HC, healthy control; L, longitudinal; MEG, magnetoencephalography; STG, superior temporal gyrus; SSD, schizophrenia-spectrum; SZ, schizophrenia; TE, echo time; TR, repetition time; ^#^, overlapping samples.

### Key insights and future directions in ultra-high field-functional magnetic resonance imaging

UHF-fMRI in psychosis has primarily leveraged quantitative improvements over lower field strengths, including greater signal to noise ratio and reduced partial volume effects relative to lower field strengths. This leads to greater sensitivity (14) in modest samples (Ns = 14–30 in Table 3) and in smaller brain regions while maintaining effect sizes comparable to those at lower field strengths (54). To date, UHF-fMRI studies have supported previous findings of both resting-state and task-based functional dysconnectivity in psychosis and linked these alterations to dysfunction over multiple scales, from the microscale to the social scale.

Though UHF-fMRI brings with it several advantages in terms of spatiotemporal resolution that benefit studies involving small brain regions and connectivity, these have not yet been widely implemented in schizophrenia. Dedicated UHF-fMRI acquisition protocols may help bring out both the quantitative and qualitative advantages of UHF in fMRI research. At the same time, as with UHF-sMRI, head motion is a major consideration for UHF-fMRI studies, as its effects on signal integrity are exacerbated at higher magnetic field strengths, which can be particularly problematic in populations with more excessive motion concerns (e.g., psychosis; (51)]. Several technological advances in MRI acquisition protocols (61, 62) and processing techniques (63) are promising in terms of mitigating these issues, and should be incorporated into future work investigating broader features at the manifest-scale (e.g., cognition) and probing longitudinal trajectories. In addition, few task-based UHF-fMRI studies have been conducted in psychosis, though task-fMRI provides a great opportunity to examine multiscale associations at all levels and particularly between the meso and manifest scales. As with most UHF studies in psychosis to date, future work with larger and more diverse samples across psychosis stages will allow for greater representativeness and examination of broader questions related to sensitivity and specificity of findings.

## Longitudinal applications of ultra-high field neuroimaging in psychosis

Psychiatric symptoms fluctuate over time as do their underlying neurobiological markers, which underscores the importance of longitudinal multiscale investigations. Though the relative novelty and limited accessibility of UHF scanners have hindered acquisition of longitudinal data, three longitudinal studies have been published to date. These studies primarily focused on metabolite variations between first-episode psychosis patients and healthy controls using UHF-MRS.

Jeon et al. (64) examined astroglial integrity using MRS indexed by myo-inositol concentration in the ACC in first-episode psychosis patients before and after the first 6 months of treatment as well as healthy controls. Astrocytes serve many roles such as maintaining reductive-oxidative processes, synaptic integrity, and myelination along with removing extracellular glutamate from the synaptic cleft space. However, it is challenging to perform *in vivo* imaging of astrocytes, hence the use of myo-inositol as a biomarker to assess astroglial activity. At baseline, FEP patients showed reduced myo-inositol concentration in the ACC relative to controls that normalized after treatment, indicating dynamic astroglial processing in FEP and supporting myo-inositol as a biomarker of psychosis.

Continuing redox imbalances, glutathione and glutamate have been observed to improve early intervention outcomes among patients that are treatment resistant to dopamine-blocking antipsychotics. Dempster et al. (28) assessed baseline glutathione and glutamate levels in the dorsal ACC, reporting no significant differences between patients and controls. However, higher glutathione levels were associated with faster treatment response, suggesting that treatments targeting glutathione levels may improve outcomes of early intervention in first episode psychosis. Jeon et al. (2) investigated the relationship between abnormal glutamatergic neurotransmission and illness trajectory in schizophrenia, but observed no significant progressive change of glutamate levels in the dorsal ACC over a 6-month period.

Increasing availability of UHF scanners along with pulse sequence and coil developments will facilitate longitudinal studies assessing structural, functional, and chemical metrics in the pathophysiology of schizophrenia across various illness stages. Collaborative efforts to harmonize and acquire 7T data across the emerging global centers will be critical to account for relatively small sample sizes reported in the current literature.

## Conclusion

This review highlighted key findings, insights, challenges, and future directions of studies to-date of psychosis using UHF neuroimaging. Due to its increased SNR, resolution, and sensitivity, UHF neuroimaging shows promise in uncovering novel neurobiological mechanisms underlying psychiatric disorders and disentangling findings derived from lower field strengths. With the rapid increase in popularity and interest in applying UHF in psychiatric and neurocognitive disorders, there is an expectation for the sophisticated modality to provide new or confirm existing findings at lower field strengths with concrete biomarkers for psychosis. Replication of robust findings at 3T, such as hippocampal volume reductions in schizophrenia and associations with symptoms, demonstrate that potential artifacts at UHF can be addressed. UHF studies that judiciously combine multiple scales of measurements from live subjects are providing greater insight into the cascading effects of dysconnectivity from the synapse-to-the-streets, clarifying multiple manifestations of psychosis. Expanding UHF multiscale studies to combine structural and functional imaging with neuro-metabolite studies will allow researchers to target as-of-yet undetectable metabolites and gather evidence of metabolite imbalances at the brain network level. Incorporating these studies with behavioral and experiential measures at the manifest and social scales will facilitate translational research and broader clinical applications, including precision diagnostics and treatment. UHF research in psychiatry is still in its infancy and most studies are affected by small sample sizes and limited acquisitions, yet its sensitivity in demonstrating neurochemical, structural, and functional changes in psychosis despite these limitations highlights its clinical potential.

## Author contributions

KL led preparing the review and drafted the initial version along with KK who wrote a part of the first draft. LP conceived, directed the review, and revised the manuscript. All authors approved the final draft and agreed to be accountable for the content of the work.
